# The distorted body: The perception of the relative proportions of the body is preserved in Parkinson’s disease

**DOI:** 10.3758/s13423-022-02099-9

**Published:** 2022-04-20

**Authors:** Megan Rose Readman, Matthew R. Longo, Neil M. McLatchie, Trevor J. Crawford, Sally A. Linkenauger

**Affiliations:** 1 Department of Psychology, Fylde College, Lancaster University, Lancaster, Bailrigg, LA1 4YF UK; 2 Department of Psychological Sciences, Birkbeck University of London, London, UK

**Keywords:** Parkinson’s disease, Motor disorder, Body perception, Somatosensory

## Abstract

Given humans’ ubiquitous visual experience of their own body, one reasonable assumption is that one’s perceptions of the lengths of their body parts should be accurate. However, recent research has shown that large systematic distortions of the length of body parts are present in healthy younger adults. These distortions appear to be linked to tactile sensitivity such that individuals overestimate the length of body parts of low tactile sensitivity to a greater extent than body parts of high tactile sensitivity. There are certain conditions featuring reduced tactile sensitivity, such as Parkinson’s disease (PD) and healthy older ageing. However, the effect of these circumstances on individuals’ perceptions of the lengths of their body parts remains unknown. In this study, participants visually estimated the length of their body parts using their hand as a metric. We show that despite the reductions in tactile sensitivity, and potential alterations in the cortical presentation of body parts that may occur in PD and healthy older ageing, individuals with mild-moderate PD and older adults of comparable age experience body size distortions comparable to healthy younger controls. These findings demonstrate that the ability to perceive the length of one’s body parts is well preserved in mild-moderate PD.

**Supplementary Information:**

The online version contains supplementary material available at 10.3758/s13423-022-02099-9.

## Introduction

Humans receive constant visual information specifying the relative proportions of their body. For example, when looking into a mirror the length of the arm relative to the torso is apparent. Consequently, one may assume that individuals will be reliably in tune with the relative proportions of their body parts. However, this does not appear to be the case; for example, although arm span and height are approximately equal, many deem height to be longer (Dreyfuss & Tilley, 1993)

The neural information underlying the perception of body proportions appears to relate the length of body parts to their tactile sensitivity (Linkenauger et al., 2015; Longo 2017). Furthermore, as body part tactile sensitivity is related to the respective cortical representation within the somatosensory cortex (Ackerley et al., 2014; Penfield & Boldrey, 1937), the perception of our body proportions appears to be related to the cortical representation of the body part in the somatosensory cortex (Linkenauger et al., 2015).

The cortical representation of body parts in the somatosensory cortex is heterogeneous (Mancini et al., 2014; Weinstein, 1968). Specifically, there is a relative magnification of cortical area devoted to body parts recruited in complex actions (e.g. the hands; Reed, & Ziat, 2018). Furthermore, as body parts with larger cortical representation display a higher tactile acuity (Reed & Ziat, 2018), tactile sensitivity is not homogenous across the body (Weinstein, 1968).

Heterogeneous tactile sensitivity influences perceptions of tactile size in that the distance between two points is perceived to be greater when the points span a region of high tactile sensitivity, for example the palm, than when they span a region of low tactile sensitivity, for example the forearm (Weber’s Illusion; Weber, 1834). Furthermore, objects of the same size are perceived to be larger when placed on a region of higher tactile sensitivity (Anema et al., 2008; Weber, 1834). However, the magnitude of Weber’s illusion experienced is substantially less (approximately 10%) than would be anticipated if perceived tactile distance was solely derived from tactile sensitivity (Taylor-Clarke et al., 2004). Consequently, the perceptual system must be employing a mechanism that preserves tactile constancy.

One potential account proposes that distorted cortical representations are rescaled according to the visually specified size of the body parts (Taylor-Clarke et al., 2004). Corroborating this, merely seeing the hand significantly reduces the perceived size of tactile stimuli (Longo & Sadibolova, 2013). Alternatively, the ‘reverse distortion’ hypothesis (Linkenauger et al., 2015), asserts that individuals perceive less sensitive body parts to be disproportionately larger than more sensitive body parts to a magnitude that offsets most of Weber’s Illusion. Based on this account (a) less sensitive body parts will be overestimated more, and (b) given equal sensitivity, larger body parts will be distorted less (Linkenauger et al., 2015). Supporting this hypothesis, when estimating the length of their body parts using their hand as a metric, participants overestimate the length of the torso, a body part of low tactile sensitivity (Mancini et al., 2014; Weinstein, 1968), the most, and the foot, a highly sensitive body part (Mancini et al., 2014; Weinstein, 1968), the least (Linkenauger et al. 2015; Linkenauger et al., 2017; Sadibolova et al., 2019).

While it is important to ascertain how healthy individuals perceive their body size, we must also consider clinical conditions that include altered tactile sensitivity such as Parkinson’s disease (PD). Although PD is considered to be a paradigmatic movement disorder (Politis et al., 2010), alterations in tactile sensitivity have been observed in PD. For example, increases in two-point tactile discrimination thresholds (Nolano et al., 2008; Schneider et al., 1987), tactile temporal discrimination thresholds (Artieda et al., 1992) and groove width required to distinguish grating orientation (Sathian et al., 1997), relative to age-matched controls have been observed in PD. If perceived body size is distorted as a function of tactile sensitivity, then we may anticipate that the perceived lengths of one’s body parts may be altered when tactile sensitivity is altered. Therefore, we may anticipate that people with PD’s perceptions of the relative lengths of their body parts may be different from healthy younger and older adults.

These reductions in tactile sensitivity may arise from the significant loss of peripheral epidermal nerve fibres, Meissner corpuscles, and free encapsulated nerves observed in PD (Nolano et al., 2008). Prior research has shown that reducing inflow from peripheral nerves in the hand to the somatosensory cortex results in increases in perceived finger size (Gandevia & Phegan, 1999). Therefore, it may be that reductions in peripheral nerve fibres lead to altered perception of body proportions in PD.

Furthermore, alterations in motor ability have been shown to influence the somatosensory cortical representation of body parts. For example, hand immobilisation results in impaired tactile perception and reduced cortical activation of the corresponding hand representation in the somatosensory cortex (Lissek et al., 2009; Weibull et al., 2011). Furthermore, expansion of cortical representations have been observed following long-term learning in the left hand of string players (Elbert et al., 1995) and in the reading finger of Braille readers (Pascual-Leone et al., 1993; Pascual-Leone & Torres, 1993). As the perception of our body proportions are related to the respective somatosensory cortical representation (Linkenauger et al., 2015; Longo 2017), altered motor ability may influence body perception in PD. Corroborating this, Bassolino et al. (2015) observed that, following 10 h of overuse, individuals perceived the arm to be longer.

Individuals’ with PD often display a greater reliance on visual information relative to other (e.g., somatosensory) information (Halperin et al., 2021; Yakubovich et al., 2020). Given that visual information alters the perceived size of tactile stimuli (e.g., Longo & Sadibolova, 2013), an increased reliance on visual information specifying the relative proportions of one’s body may mitigate the influence of altered tactile information on the perception of one’s body proportions. Under these circumstances we may anticipate that individuals with PD will display the same systematic distortions as young healthy controls.

Throughout healthy ageing reductions in tactile sensitivity (Kenshalo, 1986; Thornbury & Mistretta, 1981; McIntyre et al., 2021), and an increase in spatial thresholds (Sathian et al., 1997), coupled with a decrease in the density and distribution of touch receptors in the skin (Stevens & Patterson, 1995; Wickremaratchi & Llewelyn, 2006) have been observed. Therefore, healthy ageing may also influence perceived body proportions.

To explore the potential influence of PD and healthy ageing on individuals’ perceptions of the relative proportions of the body, individuals with mild-moderate PD, healthy older and younger adult controls estimated the length of various body parts using their hand as a metric.

## Method

### Participants

G*Power software (Faul et al., 2007) was used to perform an a priori power analysis to ascertain the sample size required to achieve adequate power. The required power (1- β) was set at .80 and the significance level (α) was set to .05. Linkenauger et al. (2015) used the same methodology as employed here to analyse the influence of tactile sensitivity (using the hand as a metric vs. a piece of dowel) on perceived body proportions; as we too are comparing groups whose tactile sensitivity may differ, we modeled anticipated effect size on the results obtained by Linkenauger et al. (2015, Experiment 1). Due to this, we anticipated a medium effect size of f = 0.6. For the frequentist parameters defined, a sample size of N = 9 is required to achieve a power of .80 at an alpha of .05.

Thirty healthy young controls (21 females), 30 healthy older adult controls (17 females), and 30 individuals (11 females) with mild-moderate PD participated. Here the exclusion criteria applied to both individuals with PD and healthy controls were those who had a diagnosis of any cognitive or additional neurological conditions beyond PD. Furthermore, as physical disability may itself alter body perception, individuals who presented with a physical disability were ineligible for the study. The mean age between the healthy older adult controls and PD patients did not differ (*t*(58) = -1.131, *p* = .263; Bayes factors provided evidence for the null for all scale-of-effects greater than 14.2 years). Eighty-four participants were right-handed (29 healthy young controls, 27 healthy older adult controls, 28 PD), and six were left-handed (one healthy young control, three healthy older adult controls, two PD patients). All participants had normal or corrected-to-normal vision. Nine participants (five PD, three older adult controls, one younger control) reported a current or a history of a diagnosis of visual impairment, including glaucoma, red/green colour blindness, macular degeneration and convergence inefficiencies.

All participants were screened for the presence of cognitive impairment through the Montreal Cognitive Assessment (MOCA; Nasreddine et al., 2005). As this study was completed virtually, due to the COVID-19 pandemic, a condensed version of the MOCA was completed. Although an abbreviated telephone version of the MOCA, excluding only visual elements, is available, completion of this version of the MOCA requires the participants to state the location of the research group. As the research group conducting this study function out of multiple locations, the research team deemed it appropriate to also remove the orientation questions relating to the location of the research lab. The normal range cut-off point for the entire MOCA is ≥ 26 out of 30 (86.66%) and the telephone-abbreviated MOCA is ≥ 19 out of 22 (86.3%). Transposing this to the subset used within this study (20 questions), the cut-off was set at ≥ 17 (85%). Following this exclusion criterion, two PD patients’ data were excluded prior to analysis. Average MOCA scores did not significantly differ between groups (*F*(2,84) = .902, *p* = .41; Bayes factors confirmed evidence for the null when comparing each condition for all scale-of-effects greater than 1.56). One younger control’s data were removed prior to analysis as their estimations were ±2 SD away from the means. Subsequently, data from 87 participants (28 PD, 30 healthy older adult controls, 29 young controls) were included in final analysis.

Of the 87 participants included in analysis, 16 (eight PD, four healthy older adult controls, two younger controls) reported a current or history of a diagnosis of psychiatric illnesses, including depression, anxiety and bipolar disorder. Furthermore, 14 participants reported a current or history of a diagnosis of rheumatic illnesses (ten PD patients, four older adult controls).

Parkinsonian symptoms were assessed using the motor aspects of daily living, the motor examination and the motor complications subscales of the Movement Disorder Society Unified Parkinson’s Disease Rating Scale (MDS-UPDRS; Goetz et al., 2008). Due to the virtual nature of this study, only the items pertaining to bradkykinesia and tremor of the motor examination subscale were assessed. Therefore, bradykinesia and tremor severity scores are reported separately. Furthermore, as not all aspects of the motor examination were completed, a Hoehn and Yahr stage was not calculated. Twenty-seven participants were receiving Parkinsonian medication and were tested under their normal medication regime. Eighteen participants indicated that they experience motor fluctuations, 16 of these participants stated that they were in a typical functioning ‘ON’ phase at the time of testing. Twenty patients were taking combination drugs (containing levodopa and a peripheral dopa-decarboxylase inhibitor, e.g., Madopar), 17 patients were taking a dopamine agonist (e.g., ropinirole), nine patients were taking a monoamine oxidase inhibitor (e.g., rasagiline) and three patients were taking a catechol-O-methyl transferase inhibitor (e.g., entacapone). Please refer to Table 1 for patient characteristics.

**Table 1:** Mean (SD) background and medical characteristics for the Parkinson’s disease (PD) healthy older adult control and younger control groups

Group	PD	Healthy older adult controls	Younger control
Age, y	65.07 (8.72) Range 51–85	68.00 (8.70) Range 54–86	24.14 (3.85) Range 18–34
Condensed MOCA (20 items included)*	18.54 (1.23) Range 17–20	18.50 (1.22) Range 17–20	18.86 (.915) Range 17–20
Years since diagnosis	5.65 (3.59) Range 1.5–16		
MDS-UPDRS motor aspects of daily living	12.24 (5.37) Range 1–22		
Condensed MDS-UPDRS motor examination- bradkykinesia **	16.54 (4.86) Range 8–29		
Condensed MDS-UPDRS motor examination – tremor ***	7.75 (6.57) Range 0–18		
MDS-UPDRS motor complications	3.82 (4.19) Range 0–15		
Years on medication	5.23 (4.20) Range .08–15		
Time since last dosage of medication (min)	157.86 (139.71) Range 5–540		
Levodopa daily dosage (mg)	625.19 (651.78) Range 100–3240		

* A condensed version of the MOCA comprising 20 questions relating to memory, attention, language, abstraction, delayed recall and four out of six items relating to orientation were administered (normal cut-off point ≥ 17 (85%)

** Average overall bradykinesia score across both sides of the upper and lower body (MDS-UPRDS items included 3.4, 3.5, 3.6, 3.7, 3.8)

*** Average overall tremor score obtained from items relating to postural and kinetic tremor of hands, and overall resting tremor amplitude and frequency (MDS-UPRDS items included 3.15, 3.16, 3.17, 3.18)

This study was ethically approved both by Lancaster University and the local National Health Service research ethics committee.

### Procedure

The study procedure used here replicated the methodology used by Linkenauger et al. (2015), with the only difference being that this study was completed via video call. Participants’ video camera facilities were turned on for the duration of the study, enabling the researcher to observe their behaviour and ensure they performed the tasks correctly. To commence this session participants were screened for the presence of mild cognitive impairment, and background health measures were obtained. At this time PD patients’ parkinsonian symptoms were assessed.

Participants were asked to make a series of estimates regarding the vertical length of parts of their bodies using their dominant hand as a metric (see Table 2; e.g., how many of your hand lengths would fit into the length of your leg). Hand length was defined as the palm-wrist intersection to the longest fingertip. Participants were encouraged to be as accurate as possible and use fractions where appropriate. Participants provided one estimation for each body part. The order of estimation was counterbalanced. All body parts were defined to the participant prior to their estimation. Following estimation, participants measured the actual length of the body parts estimated. Additionally, hand length was measured. To obtain these measures, participants were asked to call upon the assistance of another individual who placed a soft tape measure over the body region. This occurred whilst the participant was engaged in the video call. Participants were provided with a detailed instruction manual, with additional pictorial representations, detailing the body landmarks that define the lengths of the body parts in question to ensure these measures were accurate.

**Table 2:** Body parts (and associated definitions) estimated by participants

Body part	Definition
Full body	From the top of head to the bottom of the heel whilst standing
Torso	From the top of the shoulder to the hip bone
Leg	From the hip bone to the bottom of the heel whilst standing
Arm	From the protrusion of the shoulder to the tip of the longest finger when the arm is outstretched
Head	From the tip of the head to the lowest point of the jawline
Foot	From the back of the heel to the tip of the longest toe

### Data analysis

Participants’ estimates of the length of their body parts with respect to the hand were initially transformed into centimetres by multiplying the body part estimate by the hand length. Following this, accuracy ratios were computed for each body part by dividing the estimated length by the actual length. Consequently, a value over 1 indicates that the participant overestimated the length of that body part, and a value under 1 indicates that the participant underestimated the length of that body part. A Greenhouse-Geisser correction was applied when analyses indicated a violation of sphericity.

To ascertain whether the same overall pattern of body proportion distortion displayed here is congruent to that in the current body of literature (Linkenauger et al. 2015; Linkenauger et al., 2017; Sadibolova et al., 2019), a repeated-measures ANOVA detailing the overall pattern of distortions was completed for each group.

To analyse the influence of altered tactile perception and motoric capabilities, observed in PD, on individual’s perceptions of their body proportions; we report a mixed analysis of variance in which group (PD, healthy older adult control, young control) formed the between-subjects measure, body part (full body, torso, leg, arm, head, and foot) formed the within-subjects measure and accuracy ratio formed the dependent measure.

As a single non-significant p-value cannot be used to infer evidence for the null hypothesis (see Lakens et al., 2020), we also report Bayes factors for all 1-df analyses. Bayes factors provide a continuous measure of evidence regarding how well the data were predicted by one hypothesis (e.g., the null; H0), relative to another hypothesis (e.g., the alternative; H1). Bayes factors were calculated using the Dienes and McLatchie (2018) R script calculator and follow Jarosz and Wiley’s (2014) thresholds in which Bayes factors between 0.33 and 3 are interpreted as weak and inconclusive, Bayes factors between 0.05 and 0.33 and between 3 and 20 as moderate evidence for the null and experimental hypotheses respectively, and Bayes factors < 0.05 and > 20 as strong evidence for the null and experimental hypotheses, respectively. The model of H1 was specified using the results of Linkenauger et al. (2015, Experiment 1), who interpreted a difference in accuracy ratios of 0.31 as evidence for the alternative hypothesis. Lastly, we report robustness regions to indicate the sensitivity of the categorical conclusions drawn from the Bayes factors to the approximate scale-of-effect used. Robustness regions are reported as *RR*(*S*, *L*), where S corresponds to the smallest scale-of-effect and L to the largest scale-of-effect that would still yield the same conclusion.

Furthermore, to examine the influence of specific disease characteristics, such as years since diagnosis, time since last dose of medication, levodopa equivalent daily dose (LEDD), tremor and bradykinesia, on participants’ estimations of the length of their body parts, we report bivariate correlational analyses with a bootstrapping correction applied.

Anxiety significantly influences individuals’ perceptions of the maximum extent to which they can perform actions (Graydon et al., 2012). Perceiving the maximum extent to which one can perform an action requires individuals to scale visual information specifying the environment to one’s morphologically dictated action capabilities (Proffitt & Linkenuager, 2013), and, therefore, is somewhat contingent upon the individual’s representation of the morphology of their body part. Therefore, it may be that anxiety and related psychiatric conditions influence individuals’ perceptions of their body proportions. Subsequently a mixed analysis of covariance (ANCOVA) was conducted to ascertain whether psychiatric conditions influenced individuals’ perceptions of their body proportions in this sample. Psychiatric conditions did not influence healthy younger controls or individuals with PD’s perceptions of their body proportions, nor did controlling for anxiety influence the differences between groups (see 14 for the full statistical analysis). However, the presence of psychiatric conditions significantly influenced the pattern of body size overestimation in healthy older adults.

## Results

### Overall body proportion distortions

#### Healthy younger controls

There was a significant main effect of body part (*F*(2.649, 74.172 = 12.707, *p* < .001, ηp^2^ =.312). Bayes factors provided moderate to strong evidence that the torso was overestimated the most (8.14 < all *B*s < 1.82 × 10^5^), and the foot the least (29.10 < all *B*s < 1.82 × 10^5^; see Table 3). The full body was overestimated more than the arm (*B*_*N(0,0.31)*_ = 7.89), but the data were inconclusive when comparing the full body with the leg (*B*_*N(0,0.31)*_ *=* 1.98) or the head (*B*_*N(0,0.31)*_ *=* 1.83). The leg was overestimated to the same extent as the head (*B*_*N(0,0.31)*_ = 0.31), but the data were inconclusive when comparing the leg with the arm (*B*_*N(0,0.31)*_ = 0.38). Participants overestimated the length of the arm and head to the same extent (*B*_*N(0,0.31)*_ *=* 0.23).

**Table 3:** Group means (and standard deviations) of estimated/actual body length accuracy ratio for each body estimates across the Parkinson’s disease (PD), healthy older adult controls and young control groups

	Healthy younger adults	Healthy older adults	PD
Body part			
Full body	1.417 (.082)	1.612 (.169)	1.302 (.344)
Torso	1.625 (.104)	1.671 (.099)	1.604 (.468)
Leg	1.292 (.070)	1.433 (.118)	1.219 (.431)
Arm	1.220 (.058)	1.320 (.082)	1.189 (.287)
Head	1.242 (.049)	1.319 (.065)	1.194 (.329)
Foot	1.031 (.032)	1.096 (.058)	1.094 (.238)

This pattern of distortions reflects that observed by Sadibolova et al. (2019), with the only exception being that we observed that participants overestimated the leg to the same extent as the arm, while Sadibolova et al. (2019) observed the reverse.

#### Healthy older adult controls

There was a significant main effect of body part *F*(2.37, 68.822) = 6.601, *p* < .001, ηp^2^ =.185). Bayes factors provided strong evidence that all body parts were overestimated more than the foot (11.78 < all *Bs* < 2.70 × 10^7^; see Table 3). There was moderate to strong evidence that participants overestimated the torso more than the arm (*B*_*N(0,0.31)*_ = 14.90) and head (*B*_*N(0,0.31)*_ = 638.12), but the evidence was inconclusive when comparing the torso to the full body (*B*_*N(0,0.31)*_ = 0.48) or leg (*B*_*N(0,0.31)*_ = 1.95). The data were inconclusive for all other comparisons (0.53 < all *B*s < 1.79) with the exception of the comparison between the arm and head, for which there was moderate evidence for the null hypothesis (*B*_*N(0,0.31)*_ = 0.29).

This pattern of distortions is comparable to that observed by Sadibolova et al., (2019), with the only deviation being that here participants overestimated the arm and leg to the same extent, whereas Sadibolova et al. (2019) observed that participants overestimated the arm more than the leg.

#### Individuals with Parkinson’s disease (PD)

There was a significant main effect of body part (*F*(3.64, 98.30) = 10.27, *p* < .001, ηp^2^ =.276). Bayes factors provided extremely strong evidence that participants overestimated the torso the most (138.77 < all *Bs* < 1.44 × 10^6^; see Table 3). There was strong evidence that participants overestimated the full body relative to the foot (*B*_*N(0,0.31)*_ = 21.10). There was moderate evidence that estimates for the leg, arm and head did not differ from one another (0.17 < all *B*s < 0.31). The data were inconclusive for all other comparisons (0.41 < all *B*s < 1.12).

This pattern of distortions parallels previous literature; however, individuals with PD overestimated the size of the head more and the arm less than previously observed (Linkenauger et al., 2015; Sadibolova et al., 2019).

### The influence of PD on the perception of body proportions of self

**Fig. 1:**
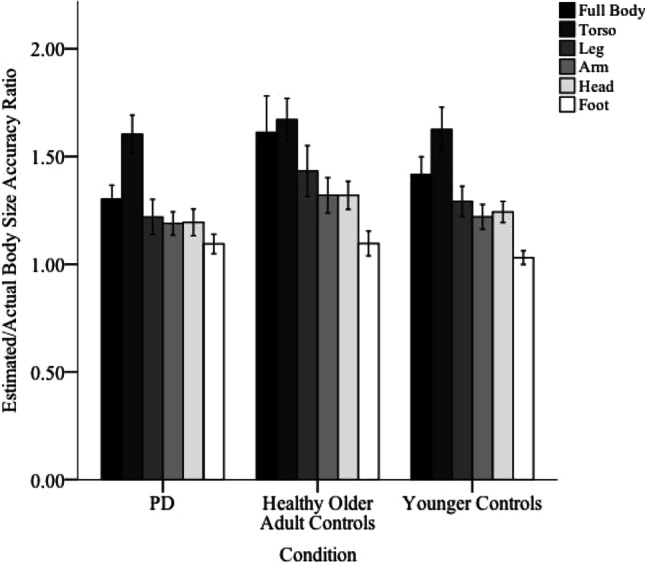
Group means of estimated/actual body length accuracy ratio for each body estimate across the Parkinson’s disease (PD), healthy older adult controls and young control groups. Error bars represent ±1 SE calculated within each condition

There were no significant differences in the accuracy of the perceived length of body parts between the PD (M_acc_= 1.267, SE_acc_ = 0.058), healthy older adult (M_acc_= 1.408, SE_acc_ = 0057), and younger control groups (M_acc_= 1.304, SE_acc_ = 0.057; *F*(2, 84) = 1.637, *p* =.201, η_p_^2^ = .038; see Fig. 1). Bayes factors provided strong evidence that PD patients and healthy older adults overestimated to the same extent (*B*_*H(0,0.31)*_ = 0.09), moderate evidence that PD patients and healthy younger adults overestimated to the same extent (*B*_*H(0,0.31)*_ = 0.28), and inconclusive evidence for the null when comparing healthy older adults and healthy younger adults (*B*_*H(0,0.31)*_ = 0.55).

### The influence of PD characteristics on self perceptions of their body proportions

Years since diagnosis, years on medication, time since last dosage of medication, the presence of tremor and motor complications were not related to the accuracy of perceived body proportions. LEDD correlated with head (*r* = .561, *p = .*004) accuracy. Overall motor aspects of daily living correlated with head *(r* = .527, *p* = .008) and arm (*r* = .413, *p* = .045) accuracy. Bradykinesia correlated with head (*r* = .514, *p* = .010), arm (*r* = .516, *p* = .010) and torso (*r* = .513, *p* = .010) accuracy (see 14).

## Discussion

Systematic distortions in the perception of the relative proportions of body parts have been observed in healthy younger adults (Longo, 2017; Linkenauger et al., 2015; Linkenauger et al., 2017; Sadibolova et al., 2019). However, the influence of altered tactile perception and motor capabilities in ageing and PD on the perception of the relative proportions of the body remains unknown.

Across all groups, the pattern of relative body size distortions paralleled previous findings (Linkenauger et al., 2015; Linkenauger et al., 2017; Sadibolova et al., 2019). This may indicate that impaired tactile sensitivity and motor function do not alter the distortion in the perception of one’s body proportions. Alternatively, it may be that alterations in tactile sensitivity and motor abilities induce variability in individuals’ perceptions. In this sense, while some may overestimate the length of their body parts, others underestimate the length of their body parts. Through this, although the average may not differ from younger controls, we would expect greater variability. Inspection of group variances indicated that the variance across all groups differed only for full body (*p* < .001) and leg (*p* = .012) estimates. Consequently, it is unlikely that the results are an artefact of variability, and rather reflect the preservation of body distortions in PD and healthy older adults.

Reduced bradykinesia was associated with more accurate perceptions of the relative lengths of the head, arm and torso in individuals with PD. However, as all individuals with PD recruited here had mild-moderate PD, additional studies recruiting individuals with more advanced PD are required to fully decipher the influence of clinical characteristics.

As we analysed the influence of altered tactile perception and motor capabilities on the perception of the *relative* length of ones’ body parts, the data analysed are reflective of the ratio of hand length to the length of each body part. Subsequently, the conclusions only follow if hand size perception is the same across all participants. It is, however, possible that individuals with PD and healthy older adults perceive their hand size or entire body as smaller or larger than controls, and this was not captured. Subsequently, additional studies analysing absolute body size perception (e.g., Longo & Haggard’s (2010) implicit hand map methodology or comparison of body lengths to a visual standard, e.g., Slade & Russell (1973)) are required to ascertain whether absolute hand and body size perception is also preserved. Moreover, whilst this work relates body size perception to tactile sensitivity, no direct assessment of tactile sensitivity occurred. Therefore, studies that directly relate measured tactile sensitivity to the perception of the relative lengths of body parts are required to confirm this link.

The observed preservation of the perceptual abilities is based on visual judgements. Therefore, whilst visually guided perceptions may remain unaffected, analysing body perception via alternative channels may reveal a different picture. In this sense although we did not observe an effect of age, previous studies have found significant alterations of body representation in ageing through the landmark localization task (Sorrentino et al., 2021). Similarly, neurotypicals embody tools and alien limbs within their body representations (e.g., Garbarini et al., 2015); however, this ability appears impaired in PD (Scarpina, et al., 2019).

It may be that the observed reductions in tactile sensitivity are not paralleled with alterations in the somatosensory cortex. Specifically, as alternations in tactile perception have been observed in the fingers (Nolano et al., 2008; Schneider et al., 1987), forearm (Sathian et al., 1997), thigh (Nolano et al., 2008), leg (Nolano et al., 2008) and foot (Prätorius et al., 2003), it appears that tactile sensitivity is globally reduced in PD. Consequently, the somatosensory cortical representation of all body parts may be altered uniformly, and so preserving the topographical representation of the body. If body size perception reflects the inverse of the representation of body parts within the somatosensory cortex (Linkenauger et al., 2015), under these circumstances alterations in body perceptions will not occur. However, future research analysing clinical circumstances in which localised alterations in tactile sensitivity is required to confirm this postulation.

Dopaminergic medications are the first-line treatment for PD (Dorszewska et al., 2014; Rogers et al., 2017). Whilst initially these medications offer vast reductions in symptoms (Marsden & Parkes, 1977), following several years of levodopa therapy around 50% of patients experience fluctuations in their motor capabilities (Dupont et al., 1996), known as the *on-off phenomenon* (Bhidayasiri, & Tarsy, 2012). During ‘on’ times individuals can perform motoric actions as normal; however, during ‘off’ times the individual’s ability to perform motor actions is severely compromised (Calne et al., 1996; Lees, 1989).

Although some research indicates that altered motor ability influences cortical representations within the somatosensory cortex (Lissek et al., 2009; Weibull et al., 2011), these effects occur following prolonged alterations in motoric abilities. For example, reduced cortical activation of the hand representation has been observed following 4–10 weeks (Lissek et al., 2009), 3 days (Weibull et al., 2011) and 10 h of hand immobilisation (Avanzino et al., 2011; Avanzino et al., 2014; Bassolino et al., 2014). Moreover, the area of cortical alteration correlates with the duration of motor disruption (Liepert et al., 1995). Antiparkinsonian medication reduces motor fluctuations (DeMaagd & Philip, 2015; MacMahon et al., 1990), therefore most individuals with PD typically do not experience severely reduced motoric abilities for prolonged periods (Nutt et al., 1984). Specifically, here 50% of participants reported they had no on/off time, 30% spent ≤ 25% of their waking hours in an ‘off’ state, 14% spent 26–50% of their waking hours in an ‘off’ state, and 6% spent 51–75% of waking hours in an ‘off’ state. As individuals with PD motor capabilities are typically not severely reduced for prolonged periods, the cortical representation of the respective affected body part may not be altered.

Alternatively, individuals with PD may employ compensatory mechanisms to preserve their perceptions of their body proportions. For example, dopaminergic medication somewhat normalises tactile perception in PD (Conte et al., 2010; Lee et al., 2005; Lyoo et al., 2012; Shin et al., 2005). Thus dopaminergic medication may normalise individuals’ body proportion perceptions.

Moreover, we found that the presence of psychiatric conditions significantly influenced the pattern of body size overestimation in healthy older adults but not in individuals with PD or younger adults. Some evidence suggests that dopamine receptor deficiencies are associated with depression and anxiety (e.g., Leggio et al., 2013; Moraga-Amaro et al., 2014). Dopaminergic medications used to treat PD mitigate dopamine receptor deficiencies by effectively replacing lost dopamine (Gandhi & Saadabadi, 2021). Therefore, it may be that dopaminergic medication also protects the individual’s perceptions from the influence of the presence of psychiatric conditions.

Individuals with PD place greater reliance on visually specified information compared to other information (Halperin et al., 2021; Yakubovich et al., 2020). This reliance upon the visually specified lengths of their body parts may somewhat mitigate the influence of altered tactile sensitivity when judging the relative proportions of one’s body parts. However, future research analysing eye-movement fixation patterns, whilst estimating the relative proportions of their body parts, are required to support this assumption.

In summary, this study demonstrated that despite the reductions in tactile sensitivity and motoric capabilities, the perceptions of individuals with mild-moderate PD of the relative lengths of their body parts are similar to that of healthy older and younger adults. Appendix Table 4

### Supplementary Information

ESM 1 (SAV 32 kb)

## Appendix A

### Analysis of covariance – The influence of anxiety on the perception of body proportions

**Healthy younger adults**

Analysis revealed that there was no significant interaction between body part estimate and the presence of psychiatric conditions ((*F*(2.64, 71.53) = .813 *p* = .477).

**Healthy older adults**

Analysis revealed that the presence of psychiatric conditions in healthy older adults significantly influences body size perception ((*F*(2.50, 70.10) = 5.88 *p* = .002).

**Parkinson’s disease**

Analysis revealed that there was no significant interaction between body size perception and the presence of psychiatric conditions ((*F*(3.57, 92.80) = .503 *p* = .713).

**Across all conditions**

Analysis revealed that the presence of psychiatric conditions did not significantly influence body perception between the three participant groups ((*F*(2, 83) = 1.032 *p* = .173).

C. Bootstrap results are based on 1000 bootstrap samples

**Table 4:** Pearson correlations between average estimated/actual body part accuracy ratio and Parkinson’s Disease characteristics

	Full Body Accuracy	Torso Accuracy	Leg Accuracy	Arm Accuracy	Head Accuracy	Foot Accuracy
Years since diagnosis	-.080	.009	-.111	.195	.165	.092
Years on medication	-.158	-.045	-.228	.086	.037	-.058
Time since last dosage of medication (minutes)	.113	-.133	-.187	-.163	-.168	-.309
LEDD	.042	.004	-.060	.066	.561**	.361
UPDRS motor aspects of daily living	-.084	.263	-.122	.413*	.527**	.260
UPDRS Tremor	-.326	.035	.009	.114	.110	-.189
UPDRS Bradykinesia	-.011	.513*	-.190	.516**	.514*	.306
UPDRS motor complications	-.010	.075	-1.84	.207	.371	.301

** Correlation is significant at the 0.01 level (2-tailed)

* Correlation is significant at the 0.05 level (2- tailed)
